# Prevalence of silver resistance determinants and extended-spectrum β-lactamases in bacterial species causing wound infection: First report from Bangladesh

**DOI:** 10.1016/j.nmni.2023.101104

**Published:** 2023-02-23

**Authors:** Kazi Sarjana Safain, Mohammad Sazzadul Islam, Jumanah Amatullah, Mohammad Al Mahmud-Un-Nabi, Golam Sarower Bhuyan, Jakia Rahman, Suprovath Kumar Sarker, Md Tarikul Islam, Rosy Sultana, Firdausi Qadri, Kaiissar Mannoor

**Affiliations:** a Infectious Diseases Laboratory, Institute for Developing Science and Health Initiatives, Dhaka, Bangladesh; b Genetics and Genomics Laboratory, Institute for Developing Science and Health Initiatives, Dhaka, Bangladesh; c Department of Mathematics and Natural Sciences, BRAC University, Dhaka, Bangladesh; d Department of Immunology, Bangladesh University of Health Sciences, Dhaka, Bangladesh; e Department of Enteric and Respiratory Infectious Diseases, Infectious Diseases Division, International Centre for Diarrhoeal Disease Research, Bangladesh, Dhaka, Bangladesh; f Department of Biochemistry and Molecular Biology, Jagannath University, Dhaka, Bangladesh

**Keywords:** Wound infection, Silver resistance, Extended-spectrum β-lactamase, Co-occurrence, Low-income countries

## Abstract

**Background:**

The use of silver is rapidly rising in wound care and silver-containing dressings are widely used along with other antibiotics, particularly β-lactams. Consequently, concerns are being raised regarding the emergence of silver-resistance and cross-resistance to β-lactams. Therefore, this study aimed to determine the phenotypic and genotypic profiles of silver-resistance and extended-spectrum β-lactamases in isolates from chronic wounds.

**Methods:**

317 wound swab specimens were collected from tertiary hospitals of Dhaka city and analysed for the microbial identification. The antibiotic resistance/susceptibility profiles were determined and phenotypes of silver resistant isolates were examined. The presence of silver-resistance (*sil*) genes (*silE, silP, and silS*) and extended-spectrum β-lactamases (ESBL) (*CTX-M-1, NDM-1, KPC, OXA-48, and VIM-1*) were explored in isolated microorganisms.

**Results:**

A total of 501 strains were isolated with *Staphylococcus aureus* (24%) as the predominant organism. In 29% of the samples, polymicrobial infections were observed. A large proportion of *Enterobacterales* (59%) was resistant to carbapenems and a significantly high multiple antibiotic-resistance indexes (>0.2) were seen for 53% of organisms (P < 0.001). According to molecular analysis, the most prevalent types of ESBL and *sil* gene were *CTX-M-1* (47%) and *silE* (42%), respectively. Furthermore, phenotypic silver-nitrate susceptibility testing showed significant minimum-inhibitory-concentration patterns between *sil*-negative and *sil*-positive isolates. We further observed co-occurrence of silver-resistance determinants and ESBLs (65%).

**Conclusions:**

Notably, this is the first-time detection of silver-resistance along with its co-detection with ESBLs in Bangladesh. This research highlights the need for selecting appropriate treatment strategies and developing new alternative therapies to minimize microbial infection in wounds.

## Funding information

This study was partially funded by The World Academy of Sciences (TWAS), Research Grant number 17-555.

## 1Introduction

Antimicrobial resistance (AMR) has become a global health issue in recent decades [1,2]. Burn victims are particularly vulnerable to this predicament since wounds frequently provide a favourable-habitat for the colonization of microorganisms. CDC maintains that burn-centres have the highest incidence of primary bloodstream infections among all ICUs [3]. Wounds are associated with high-rates of morbidity and mortality and are known to be a cause of significant economic burden [4,5]. For instance, USA alone spends $25 billion every year to manage chronic wounds and the interest in wound consideration is expanding radically [6].

For the treatment of infections caused by microorganisms of mixed-species, depending on the wound type, topical antimicrobials are used which has been reflected in the increased usage of silver in wounds [7]. For the treatment of burns and chronic wounds, silver compounds have been used for hundreds of years [8]. Silver-coated dressings are used for traumatic wounds and silver-impregnated polymers are ordinarily utilized in clinical gadgets. Therefore, there is a chance to develop a risk of nosocomial infections in hospitals by developing resistance against silver-ions [9].

Concerns have been raised about the misuse of silver and the potential emergence of bacterial resistance to silver, particularly in clinical-settings. An increasing number of outbreaks caused by silver-resistant strains of *Enterobacterales* had been reported worldwide [9,10]. Many clinicians and scientists had addressed whether the inescapable usage of silver could prompt cross-protection from antimicrobials, as biocides are frequently recommended with other antimicrobials [11,12]. It has been reported that silver could affect AMR directly by targeting porin deficiency, thereby mediating cross-resistance to β-lactams in particular [13]. Thus, investigation of the frequency of silver-resistance is important because plasmid transfer to develop cross-resistance to β-lactam antimicrobials is a high possibility. Previous studies reported that silver-resistance genes could be present on a plasmid carrying AMR genes; particularly extended-spectrum β-lactamases (ESBLs) [14,15]. Also, high-level dissemination of ESBL-producing *Enterobacterales* in wound-infections had been reported in most regions of the world [16,17] which affects low-income countries the most [18].

Due to the misuse and overuse of antimicrobials and poor healthcare standards in Bangladesh, AMR has been increasing gradually. In the last decade, the research works conducted in Bangladesh on wound-infections were only limited to the phenotypes of AMR [19,20] and thus, there is a paucity of data regarding the resistance spectrum to silver-nitrate of wound-derived bacterial isolates and the associated genetic-profiles. The co-occurrence of ESBL and silver-resistance (*sil*) genes among bacteria is a matter of concern because this phenomenon may help foster AMR. Hence, we investigated the phenotypic and genotypic profiles of bacterial-resistance to silver. In addition, the co-occurrence of *sil* genes and ESBLs in isolates from chronic-wound specimens has been studied. To the best of our knowledge, this is the first evidence from Bangladesh.

## 2 Materials and method

### 2.1 Sample collection and microbiological analysis

This retrospective-analysis was conducted by reviewing records of chronic wound-swab samples that arrived at the Microbiology laboratory of Bangladesh Institute of Health Sciences Hospital and Shaheed Suhrawardy Medical College & Hospital at Dhaka, Bangladesh from January 2017 to March 2019. A total of 317 wound specimens were collected and analysed.

After superficial pre-cleansing of wounds with physiological saline, each specimen was collected by rotating a sterile, pre-moistened swab stick across the wound surface. Next, the swab was placed in the tube containing transport-medium and sent to the laboratory. The samples were then processed according to standard techniques. Bacterial identification and confirmation were performed by routine conventional microbial cultures and biochemical tests using standard techniques [21].

### 2.2 Antimicrobial susceptibility testing

In this study, the Clinical and Laboratory Standard Institute (CLSI) guideline was followed to profile the antimicrobial susceptibility/resistance pattern of the isolates [22]. This profiling was performed by the modified Kirby-Bauer disc-diffusion method on Mueller–Hinton agar (MHA) plates. The only exception was *Streptococcus agalactiae,* which was cultured in MHA with 5% sheep blood.

Antibiotic-susceptibility test discs in cartridges for tetracycline, trimethoprim-sulfamethoxazole, rifampicin, cefoxitin, nalidixic acid, cotrimoxazole, ciprofloxacin, azithromycin, ampicillin, cefixime, gentamicin, chloramphenicol, ceftriaxone, imipenem, piperacillin-tazobactam, penicillin, vancomycin, linezolid, colistin, erythromycin, televancin and clindamycin were obtained from Oxoid (Hampshire, UK). The cartridges were stored between 4 °C and −20 °C and allowed to come to room temperature before use. After inoculation with the isolates and placement of the disks, plates were incubated at 37 °C for 24 h and the zones of inhibition were measured.

### 2.3 Determination of MAR index

The multiple antibiotic-resistance (MAR) index was calculated as the ratio of the number of antibiotics to which an organism was resistant (a) to the total number of antibiotics used in the susceptibility testing for the specific organism (b) [23].

### 2.4 Phenotypic silver nitrate susceptibility testing

Entire isolates were subjected to MIC measurements by broth-microdilution method, in which ≥512 μg/mL was considered a clinical-breakpoint for silver-resistance [24]. Two-fold serial dilutions of silver-nitrate solution (Sigma Aldrich, USA) were prepared using deionized water, to obtain a concentration from 4 to 512 μg/mL.

### 2.5 Detection of ESBL and silver resistance genes

The isolated bacterial colonies were subjected to genomic DNA extraction using QIAamp DNA Mini Kit (Qiagen, Germany) and the manufacturer's instructions were followed. 16S rRNA positive DNA extracts were examined for the presence of resistance-genes for silver and β-lactams by PCR using primer-sets for each resistance-gene which has been described in Supplementary Table 1. T100™ thermal cycler (Bio-Rad, USA) was used for gene-specific PCR amplification. Each 10 μL PCR reaction volume contained 1 μL 10x PCR buffer, 0.3 μL 50 mM MgCl_2_, 0.2 μL of 10 mM dNTPs mixture, 0.5 μL forward and reverse primers, 0.05 μL of *Taq* polymerase, 5.45 μL nuclease-free water and 2 μL of template DNA. Each reaction underwent initial denaturation at 95 °C for 5 min, 35cycles of denaturation at 95 °C for 30 s, annealing at 55 °C, cyclic extension at 72 °C for 45 s, and final extension at 72 °C for 6 min. The amplicons were visualized under UV light after electrophoresis through 1% agarose gel stained with SYBR Safe (Invitrogen, USA) staining. Next, the PCR products were purified using a Quick PCR product purification kit (Invitrogen, USA).

### 2.6 Sequencing of ESBL and sil genes

Sanger DNA sequencing was performed using ABI PRISM software version 3.1.0. Sequencing data were analysed by Chromas Lite 2.4 software to identify the target sequence by alignment with the reference sequence. The obtained sequence was further analysed using Basic Local Alignment Search Tool.

### 2.7 Data analysis

Graphs were generated using GraphPad Prism v7 software. Cochran chi-square test was performed using http://www.openepi.com/website where the threshold for statistical significance was P < 0.05.

## 3 Results

### 3.1 Rate of isolation and polymicrobial infections

**Fig. 1:**
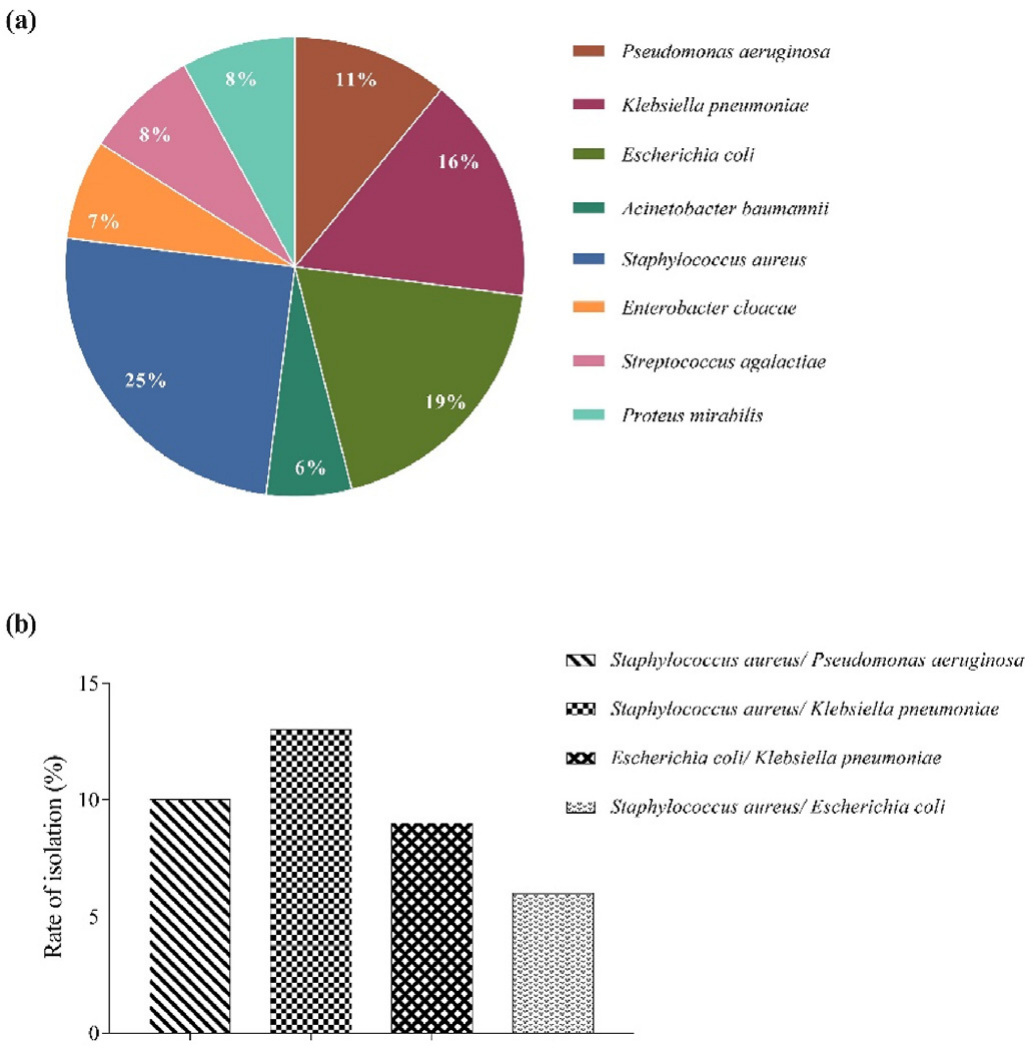
**Percentages of different species of microorganisms and polymicrobial infections.** (a) Percentages of different species of microorganisms isolated from 317 wound swab samples and (b) Percentages of the most common bacterial co-infections in the wound swab specimens.

A total of 501 strains were isolated from 317 wound-specimens. As per microbial-culture and biochemical tests, 8 different microbial species were identified which constituted of 33% Gram-positive and 67% Gram-negative organisms. The most common bacterial-species detected included *Staphylococcus aureus* (25%), followed by *Escherichia coli* (19%), *Klebsiella pneumoniae* (16%), *Pseudomonas aeruginosa* (11%), *Proteus mirabilis* (8%)*, Streptococcus agalactiae* (8%)*, Enterobacter cloacae* (7%), and *Acinetobacter baumannii* (6%) (Fig. 1a).

In addition, polymicrobial-infections were found in 91 (29%) of the infected-wounds. Two species made up the majority of polymicrobial infections; whereas three species were the highest to be isolated from a sample, and it made up 3% of the total polymicrobial infections. The most common combinations were *Staphylococcus aureus* and *Klebsiella pneumoniae* (13%), *Staphylococcus aureus* and *Pseudomonas aeruginosa* (10%), *Escherichia coli* and *Klebsiella pneumoniae* (9%) and; *Staphylococcus aureus* and *Escherichia coli* (6%) (Fig. 1b).

### 3.2 Antimicrobial resistance phenotype

Next, we wanted to see the antimicrobial-resistance patterns of the isolated Gram-negative and Gram-positive organisms (Table 1). As per antibiogram data, 67% of *E. coli* and 61% of *K. pneumoniae* were resistant to 3rd generation cephalosporins. Among *S. aureus* isolates, 73% were methicillin-resistant and 43% were vancomycin-resistant. In addition, 59% of *Enterobacterales* were resistant to carbapenem with *E. coli* (76%) and *K. pneumoniae* (69%) as the predominant microorganisms. Contrarily, *P. mirabilis* exhibited 100% sensitivity to 3rd generation cephalosporin, whereas only 9% *A. baumannii* and 10% *E. cloacae* showed resistance to 3rd generation cephalosporins. 7% *S. agalactiae* demonstrated to be penicillin-resistant.

**Table 1:** Antimicrobial resistance patterns of the organisms isolated.

Isolates	Number of tested organisms	Number of resistant organisms	% resistance among the tested organisms	Number of resistant organisms	% resistance
	Carbapenem-resistant			3rd generation cephalosporin-resistant	
***E. coli***	85	65	76	57	67
***K. pneumoniae***	65	45	69	40	61
***P. mirabilis***	40	11	27	0	0
***E. cloacae***	30	9	30	3	10
***P. aeruginosa***	50	11	22	7	14
***A. baumannii***	25	4	16	2	9
	**Vancomycin resistant**			**Methicillin-resistant *Staphylococcus aureus* (MRSA)**	
***S. aureus***	121	52	43	89	73

	**Penicillin-resistant**		
***S. agalactiae***	40	3	7

Table 2 shows the antibiotic-resistance profile and MAR-index of the indicated organisms. The proportion of isolates with MAR-index values less than 0.2 and greater than 0.2 were 47% and 53%, respectively and the difference between these MAR-values has been found to be statistically significant (P < 0.001), demonstrating wound as high-risk contaminating source favouring growth of resistant-bacteria. A large proportion of *E. coli* (73%), *K. pneumoniae* (69%), and *S. aureus* (67%) isolates exhibited MAR values greater than 0.2.

**Table 2:** **MAR index values among the identified organisms.** P values are calculated with Cochran chi-square test.

MAR Index (Range)	*E. coli* n(%)	*K. pneumoniae* n(%)	*P. mirabilis* n(%)	*P. aeruginosa* n(%)	*A. baumannii* n(%)	*S. aureus* n(%)	*E. cloacae* n(%)	*S. agalactiae* n(%)	*P* value
<0.2	23 (27)	20 (31)	25 (62)	23 (46)	17 (68)	40 (33)	22 (73)	31 (77)	<0.001
>0.2	62 (73)	45 (69)	15 (38)	27 (54)	8 (32)	81 (67)	8 (27)	9 (23)	

### 3.3 Association of silver resistance gene variants with phenotypic silver nitrate susceptibility

Next, we determined the phenotypic and genotypic profiles of *sil* genes for the collected organisms. First, we checked MIC for silver nitrate. Consistent MIC patterns were observed in *sil* gene-carrying isolates as higher MIC endpoints were observed in most cases. 65% (101/155) of the *sil* gene-bearing isolates showed to be phenotypically-resistant (MIC ≥512 μg/mL) as shown in Table 3. With acquisition of *sil* gene, 55% *K. pneumoniae*, 55% *P. mirabilis,* 75% *E. coli*, 63% *P. aeruginosa*, 80% *A. baumannii*, 62% *S. aureus* and 70% *E. cloacae* revealed higher MIC endpoints at ≥512 μg/mL, generating significant P values. Since no isolates showed MIC for silver-nitrate at 4 μg/mL, data has not been shown for MIC at this point. Albeit, most sil-negative isolates exhibited lower MICs, 17% (59/346) of the sil-negative isolates demonstrated phenotypic silver-nitrate resistance. On the contrary, *S. agalactiae* isolates showed no resistance to silver. This observation indicates that bacterial isolates which were phenotypically resistant to silver-nitrate harboured *sil* genes and the results were statistically significant compared to sil-negative bacterial isolates.

**Table 3:** **Association of the presence of silver resistance genes with the minimum inhibitory concentration of silver nitrate.** P values are calculated with Cochran chi-square test.

Organisms	Presence of sil genes	Number of isolates	Silver nitrate MIC (μg/ml), n(%)						*P* value
			16	32	64	128	256	≥512	
***E. coli* (n** = **85)**	Positive	44	–	–	2 (5)	–	9 (20)	33 (75)	<0.001
	Negative	41	5 (12)	9 (22)	–	20 (49)	–	7 (17)	
***K. pneumoniae* (n** = **65)**	Positive	22	–	–	–	4 (18)	6 (27)	12 (55)	0.008
	Negative	43	5 (12)	7 (16)	–	2 (4)	10 (23)	19 (44)	
***P. mirabilis* (n** = **40)**	Positive	11	–	–	3 (27)	2 (18)	–	6 (55)	0.001
	Negative	29	3 (10)	12 (42)	10 (34)	2 (7)	–	2 (7)	
***P. aeruginosa* (n** = **50)**	Positive	16	–	–	–	4 (25)	2 (12)	10 (63)	0.001
	Negative	34	5 (15)	12 (35)	5 (15)	9 (27)	–	3 (8)	
***A. baumannii* (n** = **25)**	Positive	5	–	–	–		1 (20)	4 (80)	0.087
	Negative	20	1 (5)	4 (20)	4 (20)	5 (25)	3 (15)	3 (15)	
***S. aureus* (n** = **121)**	Positive	47	–	–	–	8 (17)	10 (21)	29 (62)	0.001
	Negative	74	6 (8)	6 (8)	5 (7)	18 (24)	20 (27)	19 (26)	
***E. cloacae* (n** = **30)**	Positive	10	–	–	–	–	3 (30)	7 (70)	0.001
	Negative	20	2 (10)	7 (35)	3 (15)	6 (30)	2 (10)	–	
***S. agalactiae* (n** = **40)**	Positive	0	–	–	–	–	–	–	N/A
	Negative	40	6 (15)	–	15 (38)	4 (10)	11 (28)	4 (9 )	

### 3.4 Occurrence of ESBL and sil genes in bacterial isolates

The 501 isolates were further analysed for the detection of ESBL and *sil* genes by sequencing the PCR products for the listed resistance-genes (Table 4). Among the ESBL genes investigated; *CTX-M-1* (47%) and *VIM-1* (24%) were the predominant resistance-genes, followed by *KPC* (12%), *OXA-48* (9%), and *NDM-1* (8%). We found *E. coli* as the predominant reservoir of ESBLs as 49%, 38%, and 27% of *E. coli* were seen to harbour *CTX-M-1, NDM-1* and *VIM-1* genes, respectively*.* A substantially higher-proportion of *KPC* (42%) and *OXA-48* (31%) genes were observed in *K. pneumoniae* isolates. Importantly, genes encoding silver-resistance were detected exclusively in *Enterobacterales*. The most frequently identified *sil* gene was *silE* with the highest frequencies in *E. coli* (40%).

**Table 4:** Occurrence of silver resistance determinants and ESBL genes in clinical isolates.

Organisms	Percentage of antimicrobial resistance genes detected (%)							
	CTX-*M*-1	NDM-1	VIM-1	KPC	Oxa-48	silE	silS	silP
***E. coli***	49	38	27	12	28	40	19	31
***P. aeruginosa***	48	29	–	31	–	20	10	15
***K. pneumoniae***	62	19	28	42	31	23	–	23
***A. baumannii***	23	–	–	–	–	9	–	10
***S. aureus***	–	–	–	–	–	31	19	29
***E. cloacae***	39	18	–	–	–	19	9	14
***P. mirabilis***	37	–	–	–	–	41	12	39

### 3.5 Coexistence of ESBL and sil genes

**Fig. 2:**
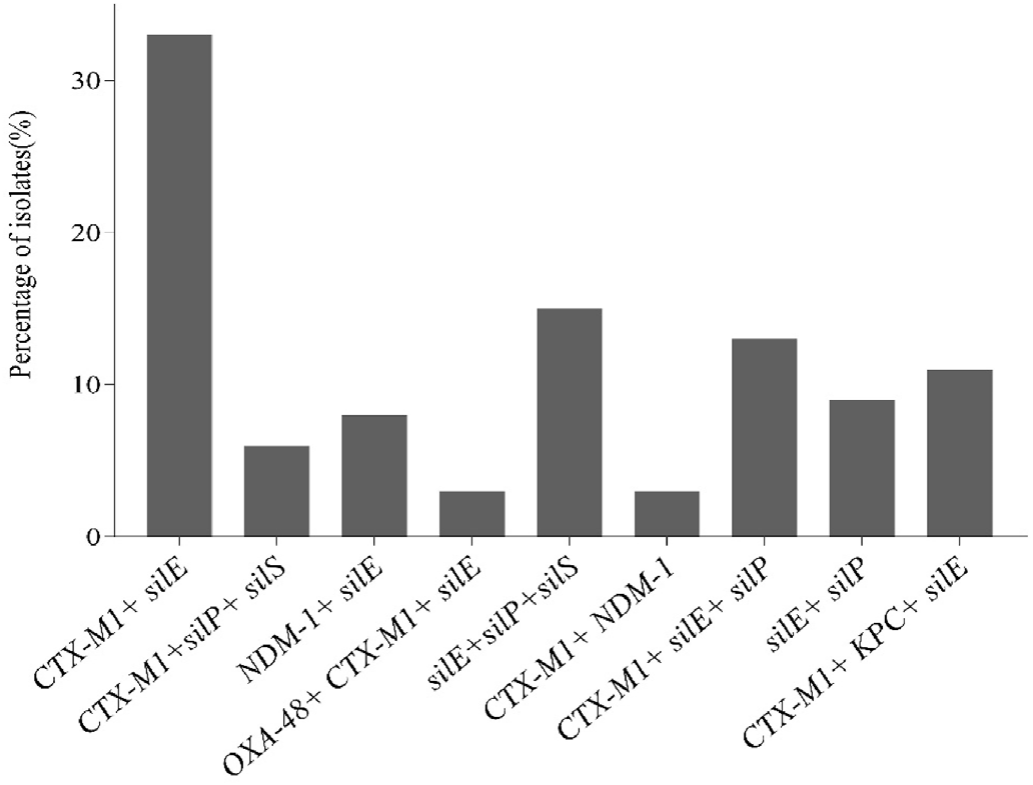
Rate of bacterial isolates harbouring multiple resistance genes.

We further demonstrated cross-resistance to silver and β-lactams and found that out of 501 clinical strains analysed, a total of 94 isolates demonstrated to harbour multiple resistance-genes (Supplementary Table 2). The most-common combinations were found to be *CTX-M-1*/*silE* (33%), *silE/silP/silS* (15%), *CTX-M-1/silE/silP* (13%), and *CTX-M-1/KPC/silE* (11%) among the 9 different types of coexistence-patterns (Fig. 2). 37% *E. coli*, 31% *K. pneumoniae,* 15% *P. mirabilis,* 13% *E. cloacae,* 20% *P. aeruginosa,* 12% *A. baumannii and* 17% *S. aureus w*ere found to harbour multiple resistance genes. *E. coli* was the prevalent type bacterium which harboured 5 different sets of resistance-genes. Overall, the data revealed that a large proportion of *Enterobacterales* (65%) harboured co-presence of single or multiple *sil* genes with or without different ESBLs.

## 4 Discussion

In recent decades, wound healing has become a major therapeutic challenge in the health-sector, since several factors play a significant role in the wound-healing process and an infected wound can result in serious complications. Furthermore, wound provides an ideal environment for the transfer of plasmids, which may contain silver and other resistance-genes, as wound biofilm has been recognized as a significant niche for plasmid transfer [25]. Although silver-resistance is gradually becoming a major concern, especially for a developing-country like Bangladesh, there is no report about the co-occurrence of silver and β-lactam resistant-bacteria in clinical-specimens from Bangladesh. This is the first study demonstrating the presence of silver and β-lactam resistant-bacteria in wound-infected patients from Bangladesh and the information may help to adopt preventive measures to inhibit the rapid spread of such resistant-bacteria within the clinical-environment.

The study detected 8 different species in the wound-specimens. In addition, polymicrobial infections were found in 29% of lesions. Such inter-species interactions are known to be dominated by bacterial-synergy, which increases their survival and complicates the eradication of infections [26]. Furthermore, more than half (59%) of the total isolates were carbapenem-resistant *Enterobacterales* (CRE) with *E. coli* (76%) as the predominant organism. This is in line with previous studies where CRE has been increasingly isolated [27,28]. In our investigation, 73% *S. aureus* was found to be methicillin-resistant (MRSA). A study identified 72% *S. aureus* strains as MRSA in an investigation in Bangladesh [29]. Therapeutic strategies for severe MRSA infections are indeed limited to fewer antibiotics and thus, it is a major health concern.

The emergence of plasmid-mediated silver-resistance raises concerns that silver-resistance will limit the efficacy of silver-based disinfectants in the future. Although some studies have reported silver-resistance [30,31], there are no such data from Bangladesh. As described by Gupta et al. [32], pMG101 is a 180-kb plasmid that accounts for resistance to multiple antibiotics and metals, including silver. The gene cassette harbouring silver resistance genes includes *silP, silA, silB, silC, silR, silS, silE, ORF105*, and *silABC* [33]. Among these, *silE, silP*, and *silS* are the most prevalent and have been investigated in prior studies [14]. Notably, our study could identify the three most common *sil* genes in Bangladesh and as expected, the occurrences of a single *sil* gene or a combination of multiple-genes were associated with increased phenotypic-resistance to silver-nitrate (higher MIC values). However, since there is no universal standard MIC breakpoint for silver-resistance, we used the cut-off value (≥512 μg/mL) according to recently published articles [10,34]. We, therefore, take this chance to emphasize the pressing necessity to establish the MIC breakpoint for silver resistance.

*CTX-M-1* and *silE* were the most frequently detected ESBL and *sil* genes, respectively. Furthermore, these genes were most frequent among *E. coli*, and 80% of *E. coli* harboured at least one of the investigated genes. *Sil* and ESBLs have most often been reported in members of *Enterobacterales*, a group of bacteria with the potential for causing infections [9]. We detected that 35% *S. aureus* that belong to ESKAPE (*Enterococcus faecium, Staphylococcus aureus, Klebsiella pneumoniae, Acinetobacter baumannii, Pseudomonas aeruginosa* and *Enterobacter* spp.) pathogens also harboured silver-resistance genes and 17% of these strains carried multiple *sil* genes. A recent study demonstrated that all plasmids from silver-resistant strains, even those from non- *Enterobacterales* group carried one of the tested replicon types of the *Enterobacterales* [35]. This along with some other studies indicate that occurrences of these resistance-genes in non- *Enterobacterales* members may have originated from organisms belonging to *Enterobacterales* group [36,37].

In addition, the frequent detection of *CTX-M-1* in the present study also justifies the fact that *CTX-M* is the most commonly distributed ESBL among *Enterobacterales* and is considered a pandemic due to its global prevalence [37]. Furthermore, 85% of the CRE were detected to be ESBL-producers, which is consistent with several other studies that have revealed the co-occurrences of multiple therapeutically relevant antibiotic-resistant genes in the same bacteria, resulting in enhanced resistance to various antimicrobials [38,39].

Many genes combined may make it easier for bacteria to emerge as high-risk enteric bacterial clones. According to multiple investigations, sil-positive isolates are substantially more common in *CTX-M-*positive isolates [15]. The study identified *CTX-M-1* and *silE* (33%) as the most prevalent combination. The observed frequent co-detection of *CTX-M-1* and *silE* appeared concordant with a recent report from India [14]. ESBL production is linked to genetic-elements such as transposons, and heavy-metal determinants, which further complicates the scenario. Furthermore, it is possible that silver may exert selective pressure on *CTX-M*-producing *Enterobacterales* [15], and hence combined actions of these genes exert broad-spectrum resistance among microorganisms.

Genetic and phenotypic silver-resistance and ESBL were observed and the existence of such genes might provide some explanation for the high wound-infection rates. This is highly worrisome in terms of spread of resistance, especially within poly-microbial-infected wounds as there are only limited therapeutic options available. Surveillance of AMR plays a major role in patient-management, not only in the developing-countries but also in the developed-countries to establish prescription-guidelines and to determine investment in new therapies. Furthermore, strong evidence shows that AMR could disseminate globally across borders [40]. So, it is high time that nations should work together in reducing the emergence of global AMR. Also, appropriate measures should be taken to prevent the spread of these resistant-bacterial isolates by increasing awareness about health and hygiene and also by restricting the random use of antibiotics and antiseptics.

This study has several important drawbacks. Sample collection sites were limited to a few tertiary hospitals in the capital of Bangladesh. Nonetheless, our findings are similar to previous studies from other nations and thus the observed results are expected to be replicable in remaining parts of the country. Along with this, a comparison of wounds that were not infected, and which became infected could also be evaluated to shed light on this issue. Undoubtedly, more studies are required to find some mechanistic explanations for silver-nitrate phenotypic discrepancy to *sil* genes and further analyse the resistant and co-resistant bacterial strains through multi-locus sequence typing and whole-genome-sequencing. Even yet, for a country like Bangladesh with limited resources, this is a challenge. We invite attention to greater investigations and discussion about this issue in different hospital-infections in this dangerous circumstance where resistant-bacterial isolates are increasing at an alarming rate.

## CRediT authorship contribution statement

**Kazi Sarjana Safain:** Data curation, Formal analysis, Methodology, Validation, Visualization, Software, Writing - original draft, Writing - review & editing. **Mohammad Sazzadul Islam:** Formal analysis, Visualization, Conceptualization, Investigation, Supervision. **Jumanah Amatullah:** Formal analysis, Methodology, Validation, Visualization. **Mohammad Al Mahmud-Un-Nabi:** Methodology, Validation, Formal analysis, Software. **Golam Sarower Bhuyan:** Formal analysis, Supervision. **Jakia Rahman:** Formal analysis, Methodology. **Suprovath Kumar Sarker:** Methodology, Software. **Md Tarikul Islam:** Formal analysis. **Rosy Sultana:** Resources. **Firdausi Qadri:** Conceptualization, Project administration, Resources, Funding acquisition. **Kaiissar Mannoor:** Conceptualization, Investigation, Project administration, Funding acquisition, Supervision, Writing - review & editing.

## Declaration of competing interest

The author declares that there is no conflict of interest that could be perceived as prejudicing the impartiality of the research reported.
