# Supplementary material for: Prevalence of silver resistance determinants and extended-spectrum β-lactamases in bacterial species causing wound infection: First report from Bangladesh
Source: New Microbes New Infect. 2023 Feb 23;52:101104. doi: 10.1016/j.nmni.2023.101104 (PMC10006487; doi:10.1016/j.nmni.2023.101104)
Supplement: Multimedia component 1 [file mmc1.docx]

**SUPPLEMENTARY TABLE 1** Primers used for amplification of different genes

| **Gene name** | **Primer name** | **Primer sequence** | **Product size (bp)** |
| --- | --- | --- | --- |
| *KPC* | KPC-1A | CTGTCTTGTCTCTCATGGCC | 795 |
|  | KPC-1B | CCTCGCTGTGCTTGTCATCC |  |
| *OXA-48* | *OXA* 48-F | GCGTGGTTAAGGATGAACAC | 400 |
|  | *OXA* 48-R | CATCAAGTTCAACCCAACCG |  |
| *VIM-1* | VIM1F | AGTGGTGAGTATCCGACAG | 241 |
|  | VIM1R | ATGAAAGTGCGTGGAGAC |  |
| *NDM-1* | NDM-F | GGTTTGGCGATCTGGTTTTC | 621 |
|  | NDM-R | CGGAATGGCTCATCACGATC |  |
| *CTX-M1* | CTX-M1-3F | AATCACTGCGCCAGTTCACGCT | 841 |
|  | CTX-M1-R2 | AGCCGCCGACGCTAATACA |  |
| *silE* | sil-E(F) | GTACTCCCCCGGACATCACTAATT | 400 |
|  | sil-E(R) | GGCCAGACTGACCGTTATT |  |
| *silS* | sil-S(F) | CTGACCTTTTTTATCAGCCTG | 741 |
|  | sil-S(F) | GCGGGTAAARACATCCTCAAT |  |
| *silP* | sil-P(F) | GGCGATAAGCTCCGCATCAGA | 524 |
|  | sil-P(F) | TCCACTTTTTCAAGACGCTCA |  |
