# Supplementary material for: Prevalence of silver resistance determinants and extended-spectrum β-lactamases in bacterial species causing wound infection: First report from Bangladesh
Source: New Microbes New Infect. 2023 Feb 23;52:101104. doi: 10.1016/j.nmni.2023.101104 (PMC10006487; doi:10.1016/j.nmni.2023.101104)
Supplement: Multimedia component 2 [file mmc2.docx]

**SUPPLEMENTARY TABLE 2** Number of strains harbouring co-resistance genes

| **Combination of resistance genes** | **Number of isolates with co-presence of resistance genes, n** | | | | | | | **Total number of isolates with co-presence of resistance genes** |
| --- | --- | --- | --- | --- | --- | --- | --- | --- |
|  | ***E. coli*** | ***K. pneumoniae*** | ***P. mirabilis*** | ***E. cloacae*** | ***P. aeruginosa*** | ***A. baumannii*** | ***S. aureus*** |  |
| *CTX-M1, silE* | 15 | 8 | 2 | - | 4 | 2 | - | 31 |
| *CTX-M1, silP, silS* | - | - | 4 | 1 | 1 | - | - | 6 |
| *NDM-1, silE* | 4 | 3 | - | - | - | - | - | 7 |
| *OXA-48, CTX-M1, silE* | - | 3 | - | - | - | - | - | 3 |
| *silE, silP, silS* | - | - | - | 2 | - | - | 12 | 14 |
| *CTX-M1, NDM-1* | 3 | - | - | - | - | - | - | 3 |
| *CTX-M1, silE, silP* | 5 | - | - | 1 | 5 | 1 | - | 12 |
| *silE, silP* | - | - | - | - | - | - | 8 | 8 |
| *CTX-M1, KPC, silE* | 4 | 6 | - | - | - | - | - | 10 |
| Total (n) | 31 | 20 | 6 | 4 | 10 | 3 | 20 | 94 |
